# Integrated physiological and transcriptional dissection reveals the core genes involving nutrient transport and osmoregulatory substance biosynthesis in allohexaploid wheat seedlings under salt stress

**DOI:** 10.1186/s12870-022-03887-0

**Published:** 2022-10-27

**Authors:** Jun-fan Chen, Ying Liu, Tian-yu Zhang, Zheng-fu Zhou, Jin-yong Huang, Ting Zhou, Ying-peng Hua

**Affiliations:** 1 School of Agricultural Sciences, Zhengzhou University, Zhengzhou, 450001 China; 2 Henan Academy of Crop Molecular Breeding, Henan Academy of Agricultural Sciences, Zhengzhou, 450002 China

**Keywords:** Core transporter, Differential gene expression, Ion homeostasis, Salinity tolerance, Triticum aestivum L

## Abstract

**Background:**

Soil salinization has become a global problem restricting the seed yield and quality of crops, including wheat (*Triticum aestivum* L.). Salinity significantly alters plant morphology and severely disrupts physiological homeostasis. Salt tolerance of wheat has been widely studied whereas core ion transporters responsive to salt stress remain elusive.

**Results:**

In this study, the wheat seedlings were subjected to salinity toxicity for morpho-physiological and transcriptomic analysis of wheat salt tolerance. There was a inversely proportional relationship between salt concentrations and morpho-physiological parameters. Under the condition of 100 mM NaCl, the H_2_O_2_, O_2_^−^, MDA content and membrane permeability were significantly increased whereas the chlorophyll content was markedly decreased. Under salt stress, a larger proportion of Na^+^ was partitioned in the roots than in the shoots, which had a lower Na^+^/K^+^ ratio and proline content. Salt stress also obviously affected the homeostasis of other cations. Genome-wide transcriptomic analysis showed that a total of 2,807 and 5,570 differentially expressed genes (DEGs) were identified in the shoots and roots, respectively. Functionality analysis showed that these DEGs were mainly enriched in the KEGG pathways related to carbon metabolism, phenylalanine, and amino acid biosynthesis, and were primarily enriched in the GO terms involving proline metabolism and redox processes. The Na^+^ transporter genes were upregulated under salt stress, which repressed the gene expression of the K^+^ transporters. Salt stress also significantly elevated the expression of the genes involved in osmoregulation substances biosynthesis, and obviously affected the expression profiling of other cation transporters. Co-expression network analysis identified *TaNHX6-D5*/*TaNHX4-B7* and *TaP5CS2-B3* potentially as core members regulating wheat salt tolerance.

**Conclusions:**

These results might help us fully understand the morpho-physiological and molecular responses of wheat seedlings to salt stress, and provide elite genetic resources for the genetic modification of wheat salt tolerance.

**Supplementary Information:**

The online version contains supplementary material available at 10.1186/s12870-022-03887-0.

## Introduction

Soil salinity, mainly referring to excessive sodium (Na) in soils, is one of the abiotic stresses that restrict a productive and sustainable development of global agriculture [1]. So far, about 1,125 million hectares of agricultural lands have been seriously threatened by salinity. Fifty percent of the land is predicted to be destroyed by salinization by the mid of the twenty-first century [2]. Salt stress severely inhibits plant growth and development through osmotic stress, ion toxicity, oxidative stress, and nutrient limitation [3, 4]. The morpho-physiological structure and metabolic balance is significantly altered under salinity toxicity, which severely reduces biomass accumulation and seed production [5].

Excessive salt in soils destroys the absorption of water by the roots, resulting in osmotic stress [6]. Under salt stress conditions, osmotic regulators, including alanine, glutamic acid, asparagine, glycine, betaine, sucrose, among others, are synthesized in large quantities. These substances are key to maintaining the balance of intracellular and extracellular osmotic pressure [7]. Previous studies have shown that plants have receptors that sense osmotic stresses, which can be converted into calcium (Ca^2+^) signals to regulate the adaptive responses of plants to salt stress [8, 9]. Generally, Na^+^ is absorbed by plant root cells mainly through non-selective cation channels (NSCC), including cyclic nucleotide-gated channels (CNGC) and glutamate receptors (GLR), as well as some high-affinity potassium (K^+^) transporters, including Arabidopsis K^+^ transporter (AKT) and high-affinity K^+^ uptake transporter (HAK) [10]. Maintaining the homeostasis of K^+^ and Na^+^ in cells are the critical factors for plant salt tolerance [11]. Excessive accumulation of Na^+^ in plants cause toxic effects on plant growth and development, such as inhibiting photosynthesis and respiration.

In order to avoid Na^+^ over-accumulation of, plants have evolved some strategies involving Na^+^ partition and sequestration to maintain ion homeostasis. The cascade signaling pathway of salt overly sensitive (SOS) comprises SOS1, SOS2, and SOS3, which are the key pathways for plant salt tolerance [12, 13]. When plants are in a high-salt environment, the intracellular Ca^2+^ concentration increases rapidly, which in turn activates the SOS cascade signal transduction pathway [14, 15]. The Ca^2+^ binding protein SOS3 interacts with the serine/threonine protein kinase SOS2 to form the SOS3-SOS2 complex, which then activates the downstream Na^+^/H^+^ antiporter SOS1/NHX7, and finally removes excessive Na^+^ from the cytoplasm [16]. SOS1 can also be directly activated by Na^+^ in the cytoplasm, independent of the SOS cascade signal transduction pathway [17]. Some of the Na^+^/H^+^ antiporters (NHXs) belong to the type of tonoplast-localized transporters responsible for vacuolar Na^+^ sequestration, and play a vital role in regulating plant salinity resistance [18, 19]. The NHX transporters use the transmembrane H^+^ electrochemical gradient produced by the tonoplast H^+^-ATPase and H^+^-PPase as the main driving force to transport excessive Na^+^ in the cytoplasm into the vacuole for Na^+^ sequestration [19]. *NHX* overexpression has been found to increase the salt tolerance of several plants species, including Arabidopsis, oilseed rape, and tomato [20–22].

The high-affinity potassium transporter (HKT) protein is another critical determinant of plant salt stress tolerance. The first *HKT* gene was cloned in wheat roots, and *TaHKT2;1* was identified as Na^+^-K^+^ co-transporter [23, 24]. *AtHKT1;1* is mainly expressed in xylem parenchyma cells and is responsible for unloading Na^+^ from the xylem, thus reducing the transportation of Na^+^ to the shoots [25]. In addition, *AtHKT1;1* is also expressed in the phloem. *AtHKT1;1* loads Na^+^ into the shoot phloem cells, and then transfers to the roots through a downward stream of the phloem, thereby avoiding shoot Na^+^ accumulation [26].

The secondary stress induced by salt stress mainly includes producing reactive oxygen species (ROS). A large amount of ROS accumulates in plant cells under salt stress, which causes severe membrane lipid peroxidation [27]. In order to prevent ROS from affecting the normal physiological processes of cells, plants have evolved a variety of ROS scavenging mechanisms, which can be roughly divided into two types: enzyme and non-enzymatic antioxidant systems. Enzymatic antioxidants mainly include superoxide dismutase (SOD), catalase (CAT), peroxidase (POD), and glutathione peroxidase (GPX), while non-enzymatic antioxidants include ascorbic acid (ASH), alkaloids, carotenoids, and flavonoids [28–32].

Wheat is one of the three essential food crops in the world, providing energy for more than 30% of the world's population [33]. The hexaploid bread wheat (*Triticum aestivum* L., 2n = 6x = 42, genome size: ∽17 Gb) is composed of three subgenomes A, B, and D. The A subgenome was contributed by *Triticum urartu*, while the B subgenome derived from the close relatives of *Aegilops speltoides*, and the C subgenome was provided by *Aegilops tauschii* [34]. The vastness and complexity of the allohexaploid wheat genome severely inhibits the elucidation of wheat salt tolerance. A comprehensive knowledge of morphological and molecular mechanisms underlying plant salt tolerance will help us to fully understand the adaptative strategies of plants to salt stress. In order to reveal the salt-stress response and adaptation related mechanisms in common wheat, therefore we were aimed to investigate the mechanism underlying wheat salinity tolerance through investigating the morpho-physiological and transcriptional responses of wheat seedlings to salt stress, and provide excellent genetic resources for molecular breeding of salinity-resistant wheat germplasm resources.

## Results

### Effects of salt stress on morphology of wheat seedlings

**Fig. 1:**
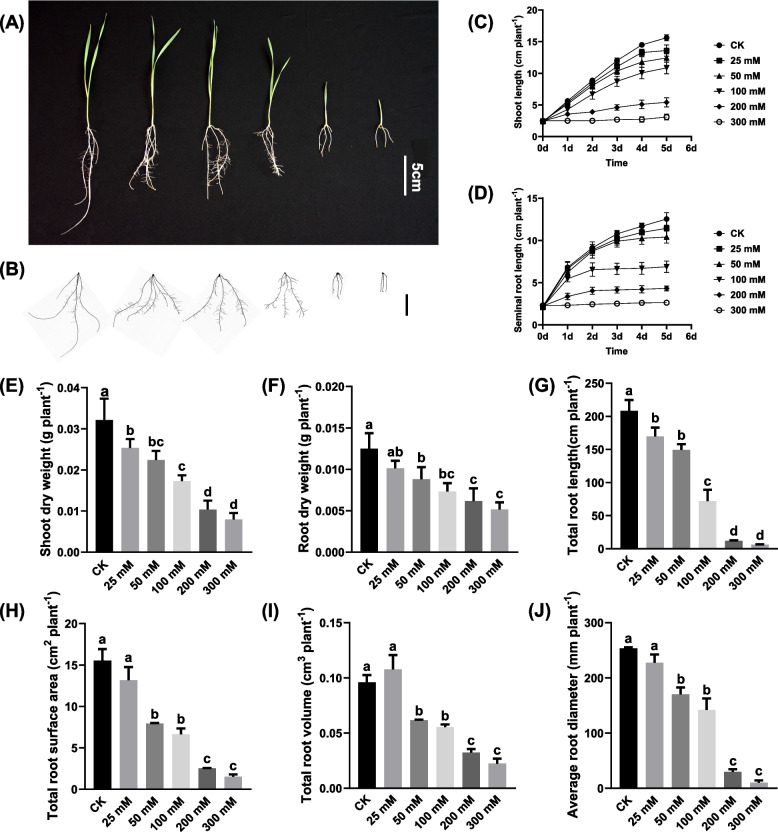
Growth status of the wheat seedlings under the control and salt stress conditions. (A) Growth status of the wheat seedlings under different salt concentrations. (B) Scanning images of wheat roots under different salt concentrations. (C–D) Dynamic wheat shoot and root lengths at different salt concentrations. (E-J) Dry weight of shoots (E), dry weight of roots (F), total root length (G), root volume (H), root surface area (I) and root tip number (J) of the wheat seedlings at different salt concentrations. After 7 d of wheat seed germination, uniform wheat seedlings were transferred to a solution containing 100 mM NaCl for 10 d. Data are mean (± SD), *n* = 5. Different letters on bar diagram indicate the statistical significance at 5% level based on Dunnett’s test

Using the hydroponic culture method, we investigated the changes in the physiological status of wheat seedlings under different NaCl concentrations (0, 50, 100, 200, and 300 mM). In this study, salt stress had an inhibitory effect on the growth of wheat seedlings; as the salt concentration increased, the inhibitory effect was gradually obvious. Compared with the wheat seedlings under control, the plants exposed to salinity showed reduced height and root length, more obviously senescent leaves, and fewer lateral roots (Fig. 1A, B). Wheat seedlings were grown under salt stress for five days; compared with the control, the 100 mM NaCl resulted in a 30.47% reduction in shoot length and 45.13% in seminal root length. The 200 mM NaCl decreased shoot length by 60.3% and seminal root length by 65.61%. The 300 mM NaCl caused an 82.28% reduction in shoot length and 79.1% in seminal root length (Fig. 1C, D). Subsequently, when the salt stress was treated for ten days, the influence of the stress on the dry weight and root morphology of wheat was analyzed. With the increase of salt concentration, the dry weight of shoots and roots gradually decreased. In addition, salt stress also significantly reduced root system architecture-related parameters, including maximum root length, total root length, root volume, root surface area, and root tip number (Fig. 1E-J). It could be concluded that the growth of wheat seedlings was severely inhibited by salt stress as seen in its growth parameters. Considering that the inhibitory roles of different NaCl concentrations on wheat biomass accumulation and root growth, we defined 25 mM and 50 mM NaCl as mild salt stress, 100 mM NaCl as moderate salt stress, and 200 mM and 300 mM NaCl as severe salt stress. In the subsequent experiments, 100 mM NaCl was used as the final concentration to explore further how wheat responded to salt stress.

### Physiological responses of wheat seedlings under salt stress

**Fig. 2:**
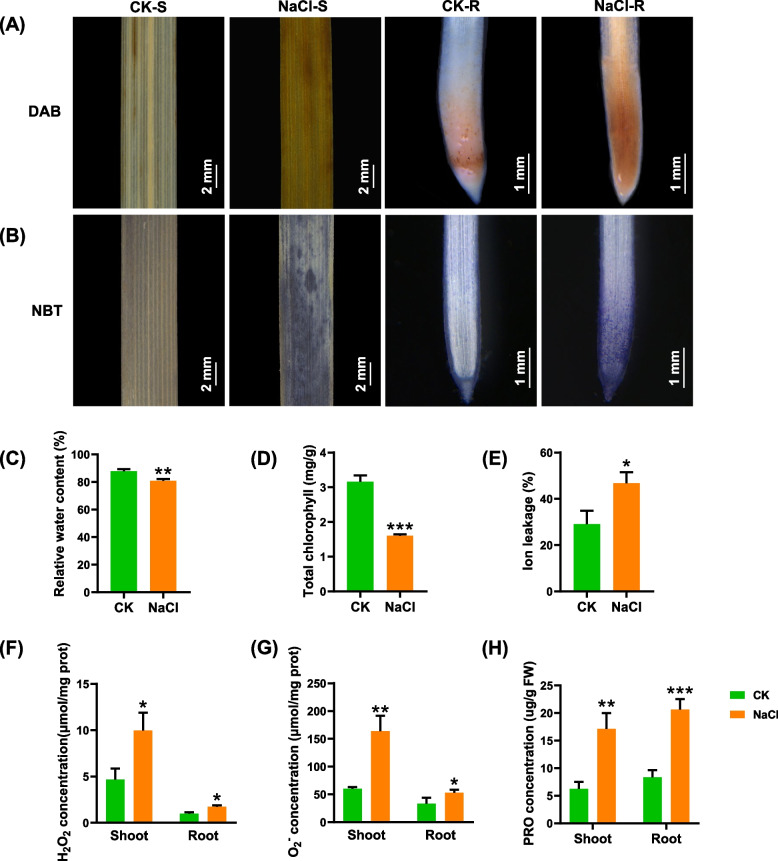
Physiological analysis of the wheat seedlings under control and salt stress. (A-B) DAB and NBT staining for reactive oxygen species in the leaves and root tips of wheat seedlings. (C-E) Relative water content (C), total chlorophyll content (D), and ion leakage rate (E) in wheat leaves under control and 100 mM NaCl conditions. (F–H) H_2_O_2_ concentrations (F), O_2_^−^ concentrations (G), and proline concentrations (H) in the shoots and roots of wheat seedlings under control and 100 mM NaCl conditions. After 7 d of wheat seed germination, wheat seedlings were transferred to a solution containing 100 mM NaCl for 10 d. Data are mean (± SD), *n* = 5. Significant differences were determined using Student’s t-test: ns, not significant; *, *P* < 0.05; **, *P* < 0.01; ***, *P* < 0.001

ROS in plant cells has a strong toxic effect on plant growth and development [27]. In order to intuitively observe the oxidation status of wheat seedlings under salt stress, we used the nitroblue tetrazolium (NBT) and diaminobenzidine (DAB) staining to detect the accumulation of hydrogen peroxide (H_2_O_2_) and superoxide anion (O_2_^−^). The darker brown spots meant the more H_2_O_2_ accumulation under salt stress compared with the control (Fig. 2A). In the presence of O_2_^−^, NBT is converted to a blue precipitate, which is distributed in or around the cell. The darker blue precipitation suggested that more O_2_^−^ accumulated under salt stress (Fig. 2B).

After exposure to salt stress, the RWC of wheat seedlings showed a 12.8% reduction in the shoots, as compared to the control (Fig. 2C). The chlorophyll content was significantly decreased, potentially resulting in a reduction in photosynthetic efficiency and inhibiting the growth of seedlings (Fig. 2D). Subsequently, we measured the ion leakage of the root system, and the ion leakage rate was significantly increased under salt stress (Fig. 2E). The H_2_O_2_ and O_2_^−^ content of the wheat seedlings were significantly increased after exposed to salinity. In detail, the content of H_2_O_2_ in the shoots and roots was increased by 54% and 47%, respectively (Fig. 2F). The O_2_^−^ content in the shoots and roots were 2.7 and 1.57 fold, respectively, compared to the control treatment (Fig. 2G). Salt stress had a obvious effect on the biosynthesis of proline in the wheat seedlings. With respect to the control, the proline content was increased by 52.9% and 60% in the shoots and roots, respectively (Fig. 2H). These results indicated that salt stress caused severely oxidative damages and destroyed cell membrane structure as well as obviously increased membrane permeability.

### Genome-wide transcriptional responses in wheat seedlings under salt stress

To understand molecular response of wheat seedlings to salt stress, we sampled the wheat seedlings, including shoots and roots, under both salt stress and control conditions, to perform genome-wide analysis of differential gene expression. High-throughput sequencing was performed on an Illumina platform; a total of 1.5 × 10^10^ nt were generated, and 1.1 × 10^8^ raw reads were obtained. The base-calling quality of Q_30_ was higher than 94.26%, and the GC content in the sequencing data of each sample ranged from 54.2% to 56.63%. More than 90% of the clean reads was aligned to the wheat reference genome of ‘Chinese Spring’ (Supplementary Table S1).

**Fig. 3:**
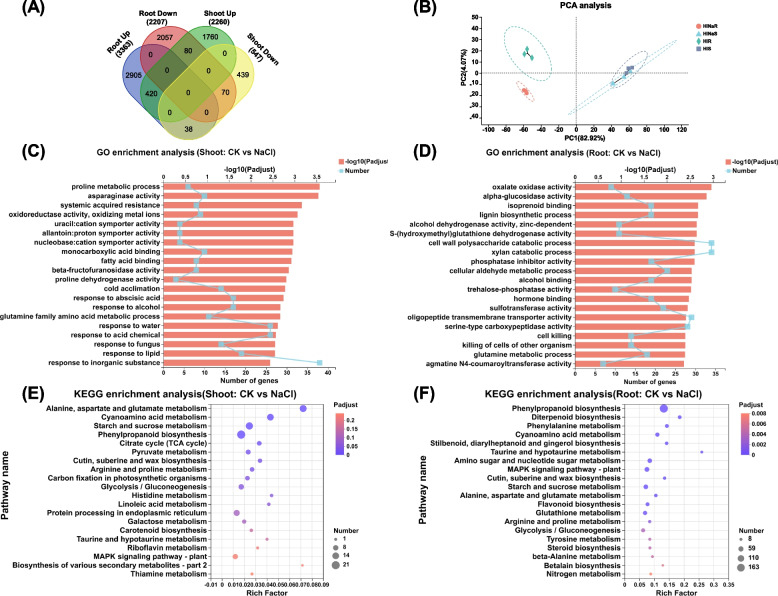
Overview of transcriptome sequencing data from wheat seedlings under control and salinity conditions. (A-B) Venn diagram (A) and principal component analysis (B) of differentially expressed genes (DEG) in the shoots and roots under control and salinity conditions. (C-D) GO enrichment analysis of the DEGs in the shoots (C) and roots (D) under control and salinity conditions. (E–F) KEGG pathway enrichment analysis of DEG in shoots (E) and roots (F) under control and salinity conditions. After 7 d of wheat seed germination, wheat seedlings were transferred to a solution containing 100 mM NaCl for 10 d

A total of 2,807 and 5,507 DEGs were detected in the roots and shoots under salt stress, respectively (Fig. 3A). In order to assess the source of variation in the overall transcriptome sequencing data, a principal component analysis (PCA) was performed. The different organs and treatments exhibited significantly different transcriptomic features (Fig. 3B), indicating organ- and condition-specific transcriptomic responses to salt stress. PCA showed that salt stress had a more obvious effect on the transcriptomic profiling in the roots than in the shoots under both salt stress and control conditions (Fig. 3B).

To have a better understanding of the DEGs, we performed a GO enrichment analysis on them. We found that most DEGs were related to plant metabolism and biosynthesis processes, such as the lignin biosynthesis process, cell wall polysaccharide catabolic process, xylan catabolic process, cellular aldehyde metabolic process, and amino acid metabolic process (Fig. 3C, D). The significantly enriched GO terms related to amino acid metabolism consisted of proline metabolic process, asparaginase activity, proline dehydrogenase activity, glutathione dehydrogenase activity, serine-type carboxypeptidase activity, and glutamine metabolic process (Fig. 3C, D). This result indicated that amino acid metabolism might play a vital role in the wheat response to salt stress. In addition, the GO items related to energy metabolisms were found to be highly enriched, such as oxalate oxidase activity, cell wall polysaccharide catabolism process, xylan catabolism process, phosphatase inhibitor activity, cellular aldehyde metabolism process, and trehalose-phosphatase activity (Fig. 3C, D). This result indicated that when exposed to salt stress, the wheat seedlings promoted sugar metabolism and provided sufficient energy for plants, thereby improving the tolerance of wheat seedlings to salt stress.

In addition, we also analyzed the metabolic pathways involving the salt-responsive DEGs in the shoots and roots of wheat seedlings under salt stress. The production of amino acids can alleviate the toxic effects of salt stress on plants. Most of the DEGs were mainly enriched in the amino acid metabolism pathways, such as phenylalanine metabolism, cyanoamino acid metabolism, alanine, aspartate and glutamate metabolism, arginine and proline metabolism, tyrosine metabolism, and histidine metabolism (Fig. 3E, F). The DEGs were also enriched in the glucose metabolism pathways, including citric acid cycle (TCA cycle), pyruvate metabolism, and glycolysis/gluconeogenesis (Fig. 3E, F). Under salt stress, the glycolysis process in plants releases a large amount of ATP, which plays a positive role in improving the stress resistance of plants. In addition, it also was enriched in the MAPK signaling pathway (Fig. 3E, F). After the plant receives the salt stress signal, the concentration of Ca^2+^ increases rapidly, transduces the signal to the protein kinase MAPK, and activates downstream genes through cascade reactions to regulate salt signal transduction, synthesis and accumulation of hormones, osmotic substances, thereby reducing the damage to plants caused by salt stress.

### Differential expression profiling of nutrient transporters in wheat seedlings under salt stress

**Fig. 4:**
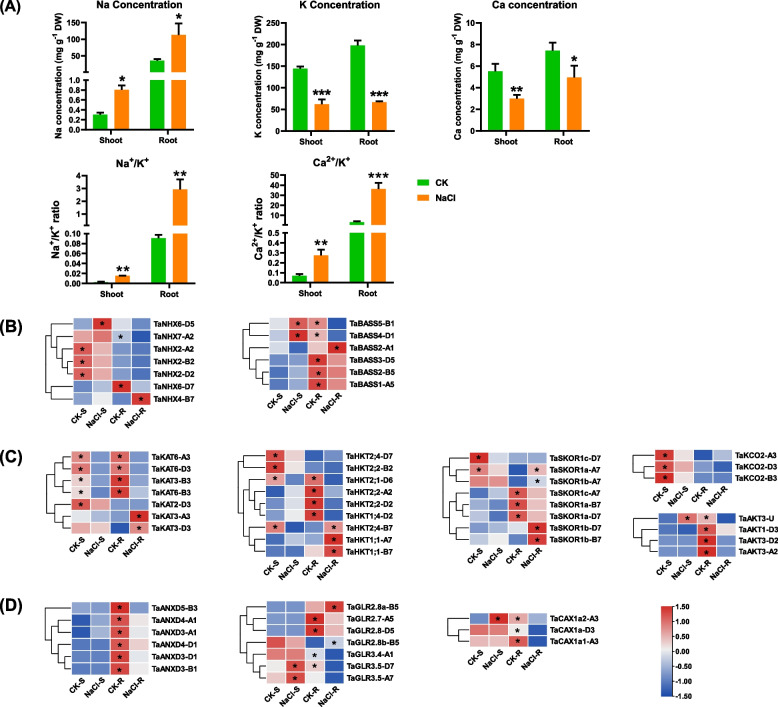
Ion concentrations of Na^+^, K^+^ and Ca^2+^ in the shoots and roots of wheat seedlings. (A) Ion concentrations of Na^+^, K^+^, and Ca^2+^, Na^+^/K^+^ ratio, and Ca^2+^/K^+^ in the shoots and roots of wheat seedlings. (B) Differential expression profiles of Na^+^ transporters. NHX, Na^+^/H^+^ antiporter; BASS, bile acid sodium symporter. (C) Differential expression profiles of K^+^ transporters. AKT/KAT, Arabidopsis K^+^ transporter; HKT, high-affinity K^+^ transporter type; KEA, K^+^ efflux antiporter; KCO, two-pore K^+^ channel; SKOR, stelar K^+^ outward rectifier. (D) Differential expression profiles of Ca^+^ transporters. ANXD, annexin D; GLR, glutamate-like receptor; CAX, cation exchanger. After 7 d of wheat seed germination, wheat seedlings were transferred to a solution containing 100 mM NaCl for 10 d. The heatmap showed the FPKM values of DEGs. Gene expression levels higher between the control and 100 mM NaCl conditions were indicated with an asterisk

To reveal the influence of salt stress on the nutrient homeostasis of wheat seedlings, we analyzed the content of Na^+^, K^+^, Ca^2+^, Mg^2+^, Cu^2+^, Fe^2+^, Mn^2+^, and Zn^2+^ in the shoots and roots under salt stress. Salt stress significantly increased the Na^+^ concentrations in both roots and shoots of wheat seedlings, while the K^+^ concentrations were decreased significantly (Fig. 4A). Genes of ion transporters play an essential role in osmotic regulation and salt stress adaptation. In order to cope with salt stress and maintain low Na^+^ and relatively high K^+^/Na^+^ in plants, the measures taken by plant cells are mainly to reduce Na^+^ uptake, increase Na^+^ efflux, and sequestrates Na^+^ in vacuoles to limit the entry of salt into plants. In this study, genes related to nutrient homeostasis deserve were more worthy of attention. NHXs are localized on the plasma membrane and tonoplast, and are mainly responsible for transporting Na^+^ from the cytoplasm to the vacuole or extracellular, reducing the cytoplasmic Na^+^ concentration. In this study, among the identified seven *NHX* DEGs under salt stress, both the tonoplast-localized *TaNHX4-B7* and the inner membrane-localized *TaNHX6-D7* were up-regulated, which indicated that salinity induced enhanced vacuolar Na^+^ compartmentation. Bile acid sodium symporter (BASS) family proteins localized in the chloroplast regulates Na^+^ influx, we found that both *TaBASS5-B1* and *TaBASS4-D1* were significantly up-regulated in the shoots under salt stress (Fig. 4B). By contrast, the expression levels of *TaBASS3-D5*, *TaBASS2-B5*, *TaBASS1-A5*, *TaBASS5-B1*, and *TaBASS4-D1* were reduced considerably in the roots (Fig. 4B).

The transcriptomic profiling showed the overall expression of stellar potassium outward rectifying channel (*SKOR*), plasma membrane-localized K^+^ influx transporter gene *AKT*/*KAT*, and high-affinity K^+^ transporter (*HKT*) on the plasma membrane was down-regulated under salt stress. *TaHKTs*, having Na^+^/K^+^ transport activity, participate in the long-distance translocation of Na^+^ in plants, and help maintain the ion balance in plants. In this study, nine *HKT* DEGs were detected in the wheat seedlings under salt stress (Fig. 4C). Among them, only *TaHKT1;1-A7*, *TaHKT1;1-B7*, and *TaHKT2;4-B7* were upregulated, promoting the transport of Na^+^ and K^+^. In this study, seven *KAT* DEGs were identified, and most *TaKATs* were downregulated in both roots and shoots, whereas *TaKAT3-A3* and *TaKAT3-D3* were upregulated in the roots, potentially promoting K^+^ absorption by plants. Based on transcriptome sequencing data, we identified eight *TaSKOR* DEGs. Except *TaSKOR1b-B7* and *TaSKOR1b-D7*, most of all the DEGs were downregulated in the shoots and roots under salt stress. In this study, all of the four *AKT* DEGs and the three *KCO* DEGs showed a downregulated pattern. These results suggested that the uptake of K^+^ by plants was inhibited by salt stress, which was consistent with the results of reduced K^+^ content tested by ICP-MS in the wheat seedlings (Fig. 4C).

Under salt stress, the concentrations of Ca^2+^ was significantly reduced in both shoots and roots of wheat seedlings (Fig. 4A). Annexin D (ANXD), cation/H^+^ exchangers (CAX), and glutamate-like receptor (GLR) transporters or channels play essential roles in regulating Ca^2+^ influx and efflux. We found that the *ANXD* family members showed much higher expression levels in the roots than in the shoots. Six *ANXD* DEGs were identified in the study, and all of them were downregulated. In this study, only ten *GRL* family members were identified to be differentially expressed under salinity. Among the *GLR* DEGs, except *TaGLR2.8a-B5* and *TaGLR3.5-D7*, the expression of other members were decreased to varying degrees. Among the genome-wide *TaCAX* family genes, we identified three DEGs, all of which were downregulated in the roots; however, only *TaCAX1a-A3* was upregulated in the shoots under high-salt conditions (Fig. 4D). The above-mentioned results indicated that the reduced expression of Ca^2+^ transporters might be mainly responsible for the decrease in the Ca^2+^ content of the wheat seedlings under salt stress (Fig. 4A).

**Fig. 5:**
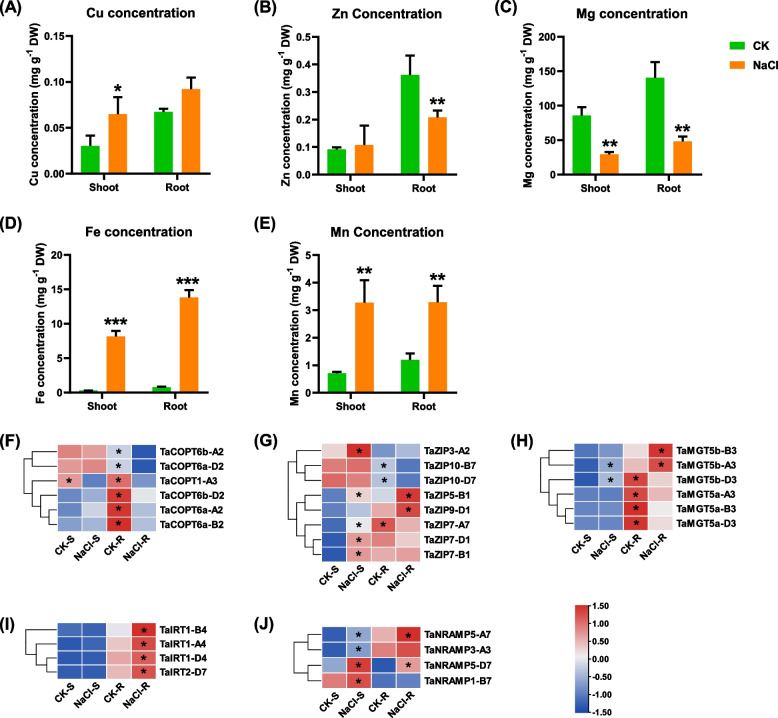
Ion concentrations of Cu^2+^, Zn^2+^, Mg^2+^, Fe^2+^, and Mn^2+^ in the shoots and roots of wheat seedlings. (A) Ion concentrations of Cu^2+^, Zn^2+^, Mg^2+^, Fe^2+^, and Mn^2+^ in the shoots and roots of wheat seedlings. (B) Differential expression profiling of Cu^2+^, Zn^2+^, Mg^2+^, Fe^2+^, and Mn^2+^ transporters. COPT, copper transporter; ZIP, ZRT/IRT-related protein; MGT, magnesium transporter; IRT, iron transporter; NRAMP, natural resistance-associated macrophage protein. After 7 d of wheat seed germination, uniform wheat seedlings were transferred to a solution containing 100 mM NaCl for 10 d. The heatmap showed the FPKM values of the DEGs. Gene expression levels higher between the control and 100 mM NaCl conditions were indicated with an asterisk

Under salt stress, the concentration of Cu^2+^ in the shoots was increased significantly, while the concentration of Cu^2+^ did not change significantly in the roots (Fig. 5A). The Copper transporter (COPT) is essential proteins that maintain the balance of copper (Cu) in cells. Six *COPT* DEGs was identified in this study, and all of them were downregulated. Notably, the expression of *TaCOPT1-A3* was repressed in both roots and shoots, as shown in Fig. 5F.

The Zn^2+^ concentration was decreased significantly in the roots under salt stress, whereas no obvious change was observed in the shoots (Fig. 5B). ZRT/IRT-related protein (ZIP) mediates the Zn^2+^ transport. A total of eight *ZIP* DEGs have been identified in the shoots and roots. All the five DEGs were upregulated in the shoots; in terms of the five *TaZIP* DEGs in the roots, *TaZIP10-B7*, *TaZIP10-D7*, and *TaZIP7-A7* were upregulated while the other two were downregulated under salt stress (Fig. 5G).

Under salt stress, the Mg^2+^ concentrations were significantly reduced in the shoots and roots (Fig. 5C). In general, the *MGT* DEGs showed higher expression levels in the roots than in the shoots. A total of six *MGT* DEGs were identified in the roots; *TaMGT5b-B3* and *TaMGT5b-A3* were significantly upregulated, while *TaMGT5b-D3*, *TaMGT5a-A3*, *TaMGT-B3*, and *TaMGT5a-D3* were downregulated (Fig. 5H).

In general, the responsive patterns of Fe^2+^ and Mn^2+^ to salt stress were similar. Under salt stress, the concentrations of Fe^2+^ and Mn^2+^ were significantly increased in both shoots and roots (Fig. 5D, E). Iron-related transporter (IRT) related to Fe^2+^ homeostasis was also responsive to salt stress. In this study, the expression of *TaIRT* DEGs was significantly higher in the roots than in the shoots, all of which, including *TaIRT1-B4*, *TaIRT1-A4*, *TaIRT1-D4*, and *TaIRT2-D7*, were significantly upregulated in the roots (Fig. 5I). NRAMP (Natural Resistance-Associated Macrophage Protein), an essential family of divalent metal transporters is involved in the absorption and transport of Mn^2+^. A total of four DEGs were identified, all of which were upregulated trend in both shoots and roots (Fig. 5J).

### Differential expression profiling of genes related to osmoregulation and antioxidants in wheat seedlings under salt stress

**Fig. 6:**
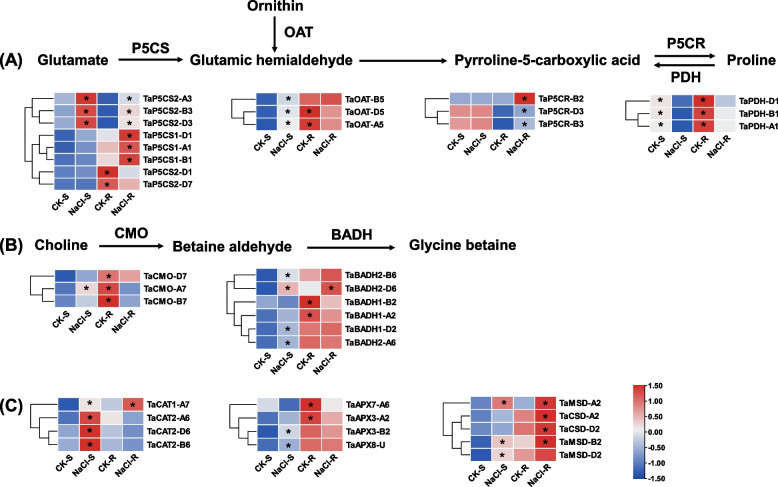
Differential expression profiling of proline and betaine biosynthesis and ROS scavenging genes in the shoots and roots of wheat seedlings under salt stress. (A) Differential expression profiling of proline synthesis pathways. OAT, ornithine aminotransferase; P5CR, pyrroline-5-carboxylate reductase; P5CS, pyrroline-5-carboxylate synthase; PDH, proline dehydrogenase. (B) Differential expression profiles of betaine synthesis pathways. CMO, choline monooxygenase; BADH, betaine aldehyde dehydrogenase. (C) Differential expression profiling of ROS scavenging genes. APX, ascorbate peroxidase; CAT, catalase; SOD, superoxide dismutase. After 7 d of wheat seed germination, wheat seedlings were transferred to a solution containing 100 mM NaCl for 10 d. The heatmap showed the FPKM values of DEGs. Gene expression levels higher between control and 100 mM NaCl conditions were indicated with an asterisk

Proline plays a role in maintaining osmotic balance under salt stress. The enzymes involved in the biosynthesis of proline consisted of pyrroline-5-carboxylate synthetase (P5CS), pyrroline-5-carboxylate reductase (P5CR), and ornithine-delta-aminotransferase (OAT). In addition, proline dehydrogenase (PDH) plays a crucial role in the degradation of proline. Under salt stress, the expression of *TaP5CS*, *TaP5CR*, and *TaOAT* genes are upregulated, while the expression of *TaPDHs* was inhibited (Fig. 6A).

Betaine is a quaternary amine osmotic substance that play an important role in salt stress tolerance through regulating the osmotic pressure inside and outside the cell. Betaine is formed by acetylcholine catalyzed by choline monooxygenase (CMO) and betaine aldehyde dehydrogenase (BADH). Under salt stress, the *TaCMO* and *TaBADH* DEGs were significantly upregulated in the shoots, whereas these DEGs, except the upregulated *TaBADH2-D6*, were downregulated in the roots (Fig. 6B). The antioxidant enzymes SOD, CAT, and APX play pivotal roles in scavenging ROS and maintaining the oxidative balance in cells. The expression of four *SOD*, four *CAT*, and five *APX* DEGs was obviously increased under salt stress. (Fig. 6C).

The responses of plants to salt stress is accompanied by the biosynthesis and accumulation of metabolites. The DEGs in the shoots and roots between the control and NaCl conditions were annotated to the metabolism overview using the Mapman software (Supplementary Figs. S2, 3), Under salt stress, many metabolic pathways have undergone significant changes, such as cell wall, sugar and acid, photosynthesis, amino acid metabolism, lipid metabolism, etc. Among them, in the cell wall metabolic pathway, genes involved in the raffinose biosynthesis were downregulated, which suggested salt stress might reduce the biosynthesis of cell wall raffinose. In the sugar-acid metabolism pathway, the genes related to the conversion of starch to sucrose were up-regulated, which suggested that the sugar metabolism pathway might be involved in the tolerance of wheat seedlings to salt stress. In addition, amino acid and lipid metabolism were highly enriched, and most of the related genes were upregulated. In addition, all the genes involving the photosynthetic response pathway were downregulated, which suggested that photosynthesis might be severely inhibited under salt stress (Supplementary Figs. S2, 3).

### Co-expression network analysis of Na^+^ transporters and the osmoregulatory substances-synthesized genes

**Fig. 7:**
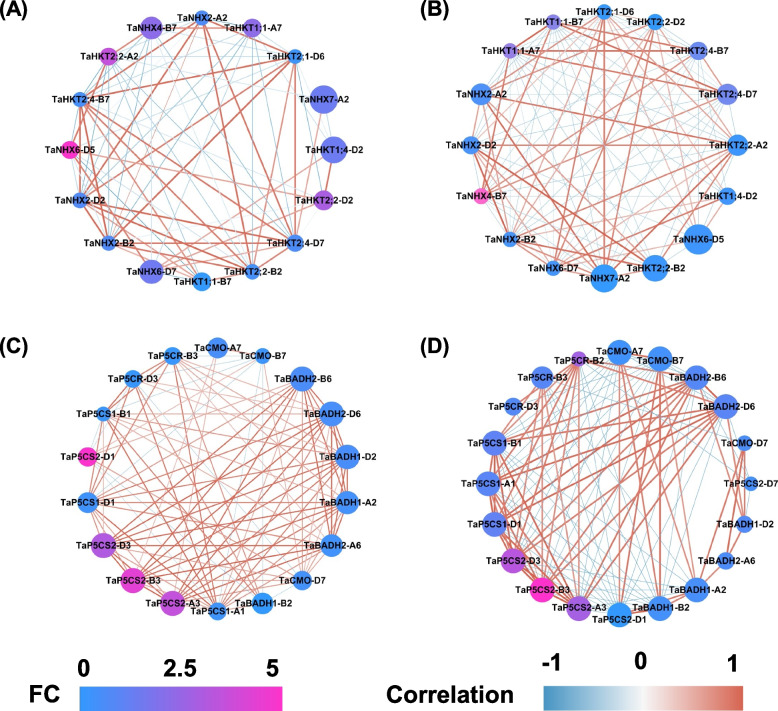
Co-expression network analysis of the *TaNHXs*, *TaHKTs*, and proline and betaine synthesis genes. (A-B) Co-expression network analysis of the *TaNHXs* and *TaHKTs* in the shoots (A) and roots (B) of wheat seedlings under salt stress. (C-D) Co-expression network analysis of proline and betaine synthesis genes in the shoots (C) and roots (D) of wheat seedlings under salt stress. Cycle nodes represent genes, and the size of the nodes represents the power of the interaction among the nodes by degree value. Edges between two nodes represent interactions between genes

In polyploid wheat, multiple-copy gene families are common; therefore, identification of the core gene(s) is a key prerequisite for the understanding of molecular mechanisms underlying important agronomy traits. Therefore, systematic analysis of plant transcriptional responses to high Cd and low Cd and molecular characterization of salt stress-responsive transporter genes will give us a comprehensive understanding of plant adaptation to heterogenous Cd conditions. Therefore, we constructed co-expression networks involving the Na^+^ transport and osmoregulatory substance biosynthesis. In terms of Na^+^ transport, seven *NHX* DEGs and nine *HKT* DEGs were selected to construct co-expression networks, which identified *TaNHX6-D5* and *TaNHX4-B7* as the core members in the shoots and roots, respectively (Fig. 7A, B). In respect to the osmoregulatory substance biosynthesis, the DEGs implicated in the biosynthesis of proline and betaine were collected for the construction of co-expression networks, which identified *TaP5CS2-B3* as a core gene regulating osmotic stress response in both shoots and roots of wheat seedlings under salt stress (Fig. 7C, D).

### Validation of the transcriptome sequencing data by RT-qPCR

**Fig. 8:**
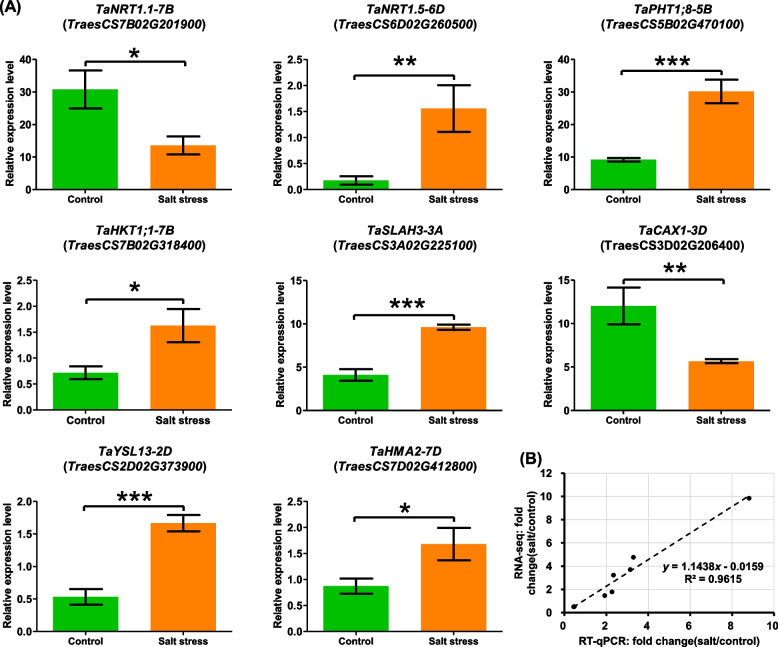
Validation of the transcriptome sequencing data by RT-qPCR. (A) Differential expression of the dual-affinity nitrate uptake transporter gene *TaNRT1.1-7B* (*TraesCS7B02G201900*), the xylem nitrate loading transporter gene *TaNRT1.5-6D* (*TraesCS6D02G260500*), the phosphate transporter gene *TaPHT1;8-5B* (*TraesCS5B02G470100*), the xylem Na^+^/K^+^ unloading transporter gene *TaHKT1;1* (*TraesCS7B02G31840*), the nitrate/chloride/anion channel gene *TaSLAH3-3A* (TraesCS3A02G225100), the cation/H^+^ exchanger gene *TaCAX1-3D* (*TraesCS3D02G206400*), the Fe^2+^ distribution transporter gene *TaTSL13-2D* (*Traes2D02G373900*), and the xylem Fe^2+^/Mn^2+^/Zn^2+^ loading transporter gene *TaHMA2-7D* (*TraesCS7D02G412800*). (B) Correlation analysis between the transcriptome sequencing and the RT-qPCR results. *, *P* < 0.05; **, *P* < 0.01; ***, *P* < 0.001

To examine the accuracy of the RNA-seq data, we selected eight DEGs to validate their expression levels via RT-qPCR assays. These genes were as follows: the dual-affinity nitrate uptake transporter gene *TaNRT1.1-7B* (*TraesCS7B02G201900*), the xylem nitrate loading transporter gene *TaNRT1.5-6D* (*TraesCS6D02G260500*), the phosphate transporter gene *TaPHT1;8-5B* (*TraesCS5B02G470100*), the xylem Na^+^/K^+^ unloading transporter gene *TaHKT1;1* (*TraesCS7B02G31840*), the nitrate/chloride/anion channel gene *TaSLAH3-3A* (TraesCS3A02G225100), the cation/H^+^ exchanger gene *TaCAX1-3D* (*TraesCS3D02G206400*), the Fe^2+^ distribution transporter gene *TaTSL13-2D* (*Traes2D02G373900*), and the xylem Fe^2+^/Mn^2+^/Zn^2+^ loading transporter gene *TaHMA2-7D* (*TraesCS7D02G412800*) (Fig. 8A). Correlation analysis showed that the gene expression was highly consistent (R^2^ > 0.96) between the transcriptome sequencing and the RT-qPCR results (Fig. 8B), which confirmed the high reliability of the sequencing data.

## Discussion

### Morphological responses of wheat seedlings to salt stress

In general, a large amount of Na^+^ and Cl^−^ in saline soil inhibits the growth of most non-halophyte plants [35]. Plant salt tolerance is a complex trait that involves multiple genes, cellular signaling pathways, and species. Salt tolerance varies greatly among different plant species. *Arabidopsis thaliana* failed to complete its life cycle at 100 mM NaCl salt concentration, while *Thellungiella halophila* is able to survive at 500 mM NaCl [36]. For cereal crops, *Hordeum vulgare* has the most salt tolerance, followed by *Triticum aestivum*, and *Oryza sativa* is the most sensitive [11].

The growth status of plants under salt stress is the most intuitive feature to assess the salt tolerance. The most common growth characteristics included plant height, fresh weight, dry weight, leaf area, and chlorophyll content [37]. Consistent with previous reports, our present study found that salt stress caused a reduction in growth performance and biomass accumulation, thus impairing the physiology and morphology of wheat seedlings (Fig. 1).

The results of this study showed that the low concentrations of NaCl (25 and 50 mM) slightly reduced the wheat seedling's growth parameters, including shoot and root dry weight, total root length, total root volume, total root surface area, and average root diameter, while inhibitory effects of 100 mM NaCl were more obvious. Exposure to high salt (200 and 300 mM) conditions induces osmotic stress that limits seedling water absorption and ion toxicity mediated by Na^+^ and Cl^−^, thereby reducing the absorption of nutrients, eventually causing plant growth to stop [38]. Plant cells require sufficient water to maintain cell turgor pressure, keeping the upright plant growth. Salt stress induces water deficiency in plant cells, causing a loss of turgor pressure that leads to wilting [39].

Chlorophyll plays a vital role in the absorption, transmission, and transformation of light energy, and its content directly affects photosynthetic efficiency [40]. Chlorophyll is the material basis of photosynthesis in plants, and its content reflects the photosynthetic efficiency of leaves [41]. In this study, the 100 mM NaCl treatment caused a significant decrease in the chlorophyll content of wheat seedlings (Fig. 2D), which may be attributed to the increase in chlorophyllase activity and chloroplast degradation [41, 42]. Salt stress results in the severe membrane destruction, increased membrane permeability, and intracellular electrolyte leakage. Moreover, the severe membrane destruction increases membrane permeability under salt stress, which further causes higher electrolyte leakage rates, which in turns aggravates the membrane damages [42]. Malondialdehyde (MDA) is the final product of membrane peroxidation, and its content reflects the level of membrane peroxidation. Therefore, MDA content and relative electrical conductivity are often used to judge the damaged degree of plant membrane systems under stresses [3]. In this study, under the treatment of 100 mM NaCl, the MDA content and relative electrical conductivity were significantly increased, which indicated that high salt damages the cell membrane structure. Salt stress directly triggers the production of reactive oxygen species in plants, and H_2_O_2_ and O_2_^−^ damage organelles and cell membranes [11]. To tolerate the osmotic stress caused by the high-salt environment, plants accumulate osmotic adjustment substances such as soluble sugar, soluble protein, and proline. Among them, proline plays an important role in maintaining cell osmotic pressure, removing ROS, and other physiological activities [43]. Many plants, such as Arabidopsis, tobacco, and *Brassica juncea*, accumulate large amounts of proline to improve tolerance when exposed to salt stress, [44–46].

### Combined morpho-physiological and transcriptional responses of wheat seedlings to salt stress

Plant roots are the first organs that directly interact with the soil, responding to biotic and abiotic stresses [3]. In this study, we found that the GO term of cell killing was enriched in the roots of wheat seedlings under salt stress (Fig. 3D). Therefore, we speculated that the cellular response of wheat seedlings was triggered by salt stress. The cell wall plays a key role in the plant salt tolerance, and limits Na^+^ and Cl^−^ enter into protoplast, thereby protecting plants from salt stress [3]. In this study, we found that the GO terms and KEGG pathways related to the biosynthesis of cell wall components were highly enriched in the shoots under salt stress (Fig. 3C, E). This result led us to further explore the pivotal components of cell wall and the regulatory genes in modulating wheat salt tolerance in our next work.

Proline accumulation is an adaptative mechanism in plants under salt stress. Our study identified the upregulation of the *P5CS*, *P5CR*, and *OAT* homologs encoding key enzymes involved in proline synthesis, and the downregulation of the *PDH* genes encoding the key enzyme implicated in proline degradation. In a previous study, abiotic stress induces the expression of *AtP5CS1*, and overexpression of *AtP5CS1* significantly increase the proline accumulation. On the contrary, reducing the expression of the *AtPDH1* gene increases proline levels. Previous studies have shown that increased proline synthesis and reduced proline degradation lead to the accumulation of proline, thereby improving the tolerance of plants to stresses [44–46]. Under salinity conditions, the characterized core genes implicated in the proline biosynthesis, including *TaP5CS2*, *TaOAT-D5*, and *TaPDH1* (Fig. 6A), might play a significant role in wheat salt tolerance. Betaine is one of the main organic osmotic regulators, and it participates in response to a variety of abiotic stresses. Stresses such as high salt, high temperature, and drought can induce an increase in the synthesis of betaine.

In plants cell, Ca^2+^ is the second messenger in plants cell; this regulates extracellular stimuli and intracellular responses, and participates in plant growth and development [47]. Under salt stress conditions, exogenous Ca^2+^ can adjust the ion balance inside and outside the cell by changing the ion channels on the Arabidopsis cell membrane, activating the enzyme activity in the cell, increasing the osmotic adjustment substances, and enhancing the ability of Arabidopsis to tolerate salt stress [48, 49]. Plant CNGCs function as non-selective cation channels, transporting primarily K^+^, Na^+^, and Ca^2+^ [50]. The expression of *AtCNGC1* and *AtCNGC13* were upregulated, while *AtCNGC3*, *AtCNGC7*, and *AtCNGC16* were downregulated under salt stress [51]. *CAXs* and *CCXs* have broad substrate specificity and transport multiple cations, such as Na^+^, K^+^, Li^+^, and Ca^2+^ [52]. *GmCAX1* is an antiporter for Li^+^, K^+^, and Na^+^, which helps maintain ion homeostasis, thereby enhancing the salt tolerance of plants [53]. *TaANXDs*, *TaCNGCs*, *TaCAXs*, and *TaCCXs* responded to salt stress and might be involved in regulating Na^+^ homeostasis under salt stress. The significant u-regulation of *TaCCXs* may be due to the decrease in intracellular Na^+^ concentrations and the increase in cytoplasmic Ca^2+^ concentrations.

Unlike K^+^, Na^+^ is not an essential element in plants. Therefore, no specific Na^+^ selective channels have been found in plants so far [54]. Due to the similar physiochemical structure of Na^+^ and K^+^, under high salt conditions, the excess of Na^+^ competes for K^+^ channel transport into the cytoplasm, resulting in K^+^ deficiency in plants [55]. *AKT1* is mainly expressed in root epidermal cells and mediates the absorption of K^+^ in the roots, whereas *AKT1* is not specifically permeable to K^+^. When the external Na^+^/K^+^ ratio is high, *AKT1* also mediates Na^+^ absorption [56]. Besides mediating K^+^ absorption, *HvHAK* in barley also mediates low-affinity Na^+^ transport [57]. The first *HKT* gene in plants is *TaHKT2;1*, which encodes a Na^+^-K^+^ co-transporter. Under salt stress, the expression of *TaHKT2;1* in wheat roots is significantly downregulated to reduce the Na^+^ content in thw roots and improve plant growth [58]. In the present study, the expression of *TaSKORs*, *TaKEAs*, *TaKATs*, *TaTPKs*, *TaHKTs*, and *TaAKTs* was significantly reduced, resulting in the significantly increased Na^+^/K^+^ ratio, thus affecting various metabolic response in plants.

In a high-salt environment, reducing the concentration of Na^+^ cytoplasmic is critical for normal plants growth and development. To cope with salt stress, plants restrict Na^+^ influx, increase Na^+^ efflux, and sequestrate Na^+^ to vacuoles [59, 60]. SOS1 is mainly expressed in the epidermis of root tip regions; under salt stress conditions, the transcription level of *SOS1*/*NHX7* is upregulated, which is considered a sensor of Na^+^ signaling and essential for cell Na^+^ efflux [12, 13]. *NHX1* sequestrates Na^+^ into vacuoles and reduces the content of cytoplasmic Na^+^, thereby enhancing the plant salt tolerance. High expression levels of *TaNHX1* expression enhance the wheat salt tolerance; in the transgenic lines, the K^+^ content is increased in the shoots, and the vacuolar Na^+^ sequestration is enhanced in the roots [61]. BASS2 is a type of the Na-dependent pyruvate transporter localized on the chloroplast membrane. The influx of Na^+^ mediated by *BASS2* is balanced by the efflux of Na^+^ mediated by *NHD1* [61].

Mg^2+^ is one of the crucial components of plant chloroplasts, and it also promotes the activation of enzyme systems. Under salt stress, the absorption of Mg^2+^ decreases, which leads to the destruction of chloroplast structure, growth inhibition, and metabolic disturbance. *OsMGT1* is an Mg^2+^ transporter localized on the plasma membrane, and previous studies have shown that *OsMGT1* is involved in the adaptive response to aluminum toxicity and salt stress [62, 63]. *OsMGT1* regulates the transport activity of *OsHKT1;5* and promotes the unloading of Na^+^ from the xylem sap [63]. In this study, the significant down-regulation of *TaMGT* expression might lead to a significant decrease in Mg content in wheat seedlings, which might further aggravate chloroplast damages.

Zn, Fe, and Cu are trace elements that play essential roles in maintaining the normal physiological metabolism of plants. This results in plant nutrient deficiency and metabolic disorders due to the high concentrations of salt competing for essential elements [64]. Under salt stress, the content of Cu^2+^, Zn^2+^, and Fe^2+^ in *Suaeda salsa* seeds were significantly decreased [65]. Under Fe deficiency conditions, salt stress induces the expression of *AtNRAMP3* and *AtYSL2* in Arabidopsis, which promotes the utilization of Fe by cell walls and vacuoles [66]. In this study, the expression levels of *TaNRAMP*, *TaZIP*, and *TaIRT* were obviously reduced under salt stress (Fig. 6), which is consistent with other previous findings that salt stress causes a nutritional imbalance in plants.

Under abiotic stresses, the types and quantities of metabolites and related metabolic pathways in plants are adjusted to tolerate the stress. Under salt stress, plants will synthesize osmoregulation substances and elevate the osmotic potential of plant cells, thereby improving the water retention capacity of cells, which is an effective way to tolerate salt stress. The enhanced metabolism of amino acids can scavenge reactive oxygen species and improve osmotic stress, which greatly enhances the ability of wheat seedlings to resist salt stress.

## Conclusions

Salt stress has become one of the abiotic stresses restricting crop yield and quality. Under salt stress, Na^+^ competes with other nutrient ion absorption transporters, which results in physiological deficiency and imbalance of nutrients in plants. Our study analysed the morphological and physiological changes in the shoots and roots under salt stress, and utilized the transcriptome data and co-expression networks to analyze the expression profiles and identified the core members of the genes related to Na^+^ homeostasis and other ion transport. In summary, these results might provide excellent genetic resources and a theoretical basis for the genetic improvement of wheat salt tolerance.

## Materials and Methods

### Plant material and growth condition

In this study, the wheat cultivar ‘Zhengmai 1860’, belonging to allohexaploid wheat (*Triticum aestivum* L., AABBDD, 2n = 6x = 42), was used as the material for salt stress treatments. ‘Zhengmai 1860’ (1000-seed weight: ~ 48.5 g), the semi-winter strong-gluten wheat cultivar derived from ‘Zhoumai 22’, ‘Zhengmai 1410’, and ‘Zhengmai 0856’, was developed at the Wheat Research Institute of Henan Academy of Agricultural Sciences (Zhengzhou, China). The plump wheat seeds were sterilized in 1% NaClO for 10 min, rinsed with sterile water several times, and finally spread on moist filter paper. After 4 d of germination, the seedlings with uniform growth were selected and transferred into the hydroponic Hoagland solution. The basic Hoagland solution containing 3.59 mM Ca(NO_3_)_2_, 8.7 mM KNO_3_, 0.713 mM NH_4_NO_3_, 1.516 mM MgSO_4_, 1.314 mM KH_2_PO_4_, 62.5 μM FeSO_4_, 44.6 μM EDTA, 48.5 μM H_3_BO_3_, 13.2 μM MnSO_4_, 1.36 μM ZnSO_4_, 0.501 μM CuSO_4_, and 2.55 μM (NH_4_)_2_MoO_4_. The wheat seedlings were cultivated in a lightroom under the following growth conditions: light intensity 500 μmol m^−2^ s^−1^, the temperature of 28℃ day/22 °C night, light 16 h light/8 h dark, and relative humidity 65%. To maintain the constant nutrient concentrations, the nutrient solution was replaced every five days.

### Root architecture system analysis

The seedlings of wheat plants after seed germination were transferred to the Hoagland solution containing 0, 25, 50, 100, 200, 300 mM NaCl for 10 d until sampling. The samples were dried in an oven at 65℃ for 72 h until constant weight to measure the dry weight of roots and shoots. The roots of fresh wheat seedlings were scanned using an EPSON scanner (EPSON perfect V700), and the roots images were analyzed with WinRHIZO Pro™ (Version 2019a, Regent Instruments, Canada) to characterize total root length, root tip number, root surface area, root volume, and average root diameter. Each sample contains three independent biological replicates.

### Histochemical staining

ROS accumulation under salt stress was detected by the NBT and DAB staining. The leaves and root tips of the wheat seedlings, grown under the control (0 mM NaCl) and salt stress (100 mM NaCl) conditions for 10 d, with 100 mM NaCl were immersed into DAB solution (1 mg mL^−1^) and NBT solution (1 mg mL^−1^) under dark for 8 h to detect the H_2_O_2_ and O_2_^−^ content. When brown and blue spots appeared, these leaves and root tips were transferred into 95% ethanol solution until chlorophyll was removed entirely. Each sample contains three independent biological replicates.

### Measurement of physiological parameters

The leaves of wheat seedlings, grown under the control (0 mM NaCl) and salt stress (100 mM NaCl) conditions for 10 d, were cut and soaked in 5 mL 95% ethanol for 48 h under dark conditions, until the leaves were completely bleached. Each sample contains three independent biological replicates. As described here, an extract absorbance was measured with a spectrophotometer and the chlorophyll content was determined according to the following formula:

Chl a = 12.7A_663_ – 2.69A_645_

Chl b = 22.9A_645_ – 4.68A_663_

Total Chl =20.2A_645_ + 8.02A_663_

The fresh leaf weight of wheat seedlings, grown under the control (0 mM NaCl) and salt stress (100 mM NaCl) conditions for 10 d, was determined immediately, and then the dry weight was measured after oven-dried at 80 °C for 72 h until constant weight. Relative water content (RWC) = [(leaf fresh weight-leaf dry weight)/leaf fresh weight] × 100%. Each sample contains three independent biological replicates.

The roots of fresh wheat seedlings, grown under the control (0 mM NaCl) and salt stress (100 mM NaCl) conditions for 10 d, were washed three times with distilled water and then soaked in 5 mL 0.4 M mannitol at 25℃ for 4 h. The solution conductivity was determined to be electrical conductivity 1 (EC1). After the roots were placed in a water bath at 85℃ for 20 min, the conductivity of the solution was determined to be EC2. Electrolyte leakage rate (%) = (EC1/EC2) × 100. Each sample contains three independent biological replicates.

The wheat seedlings, grown under the control (0 mM NaCl) and salt stress (100 mM NaCl) conditions for 10 d, were selected to determine the content of H_2_O_2_, O_2_^−^ and proline. Fine powder (∽0.2 g) of fresh wheat seedlings was incubated in 50 mM phosphate buffer, and the homogenate was centrifuged at 10,000 × g for 20 min at 4 °C. The supernatant (0.5 mL) and 0.5 mL 10 mM hydroxyl ammonium chloride was mixed thoroughly, and incubated at 37 °C for 20 min. The resultant was added to 17 mM p-aminobenzene sulfonic acid (0.5 mL) and 7 mM α-naphthylamine (0.5 mL), and was then incubated at 37 °C for 20 min, and was finally added to ethylether (0.5 mL) for 20-min incubation. The O_2_^−^ content was calculated in the shoots and roots based on the absorbance at 536 nm [67]. The H_2_O_2_ content was determined according to the method provided by Patterson et al. [68]. Fresh wheat seedlings (∽0.2 g) were triturated with pre-chilled acetone and centrifuged at 8,000 × g for 10 min at 4 °C. Then, the supernatant (1.0 mL) was mixed with ammonia (0.2 mL) and 20% TiCl_4_ (0.1 mL), and the precipitate was repeatedly washed with acetone for the 3–5 times and completely dissolved in 5.0 mL of 2.0 M H_2_SO_4_. The absorbance of all the samples was determined at 415 nm using a spectrophotometer. Each sample contains three independent biological replicates.

The proline content was determined by the ninhydrin colorimetric method [56]. The fine powder of fresh wheat seedlings, grown under the control (0 mM NaCl) and salt stress (100 mM NaCl) conditions for 10 d, was incubated in 3% sulfosalicylic acid, and was placed in 96 °C water bath for 10 min, and then cooled and centrifuged at 10,000 × g for 15 min. After that, the supernatant was mixed with acid-ninhydrin (1.0 mL) and glacial acetic acid (1.0 mL), and the mixture was incubated in a water bath at 95 °C for 1 h. After cooling, 4.0 mL toluene was added, and the absorbance was measured at 520 nm. Each sample contains three independent biological replicates.

### ICP-MS analysis of ion contents

After growing under the control (0 mM NaCl) and salt stress (100 mM NaCl) conditions for 10 d, the wheat seedlings were divided into roots and above-ground parts, then dried to constant weight. Samples were digested with a mixture of nitric acid and H_2_O_2_ and examined for sodium (Na), potassium (K), calcium (Ca), magnesium (Mg), iron (Fe), manganese (Mn), copper (Cu), and zinc (Zn) content using an inductively coupled plasma mass spectrometry (ICP-MS; NexION™ 350X, PerkinElmer). Each sample contains three independent biological replicates.

### RNA extraction for transcriptomic analysis

Total RNA of the fresh wheat seedlings, grown under the control (0 mM NaCl) and salt stress (100 mM NaCl) conditions for 10 d, was extracted using the Trizol reagent. The shoots and roots were individually sampled, and each group included three independently biological replicates for transcriptome sequencing. Genomic DNA was eliminated using TaKaRa DNase I, and oligo(dT) magnetic beads were used to purify mRNAs that was then subjected to fragmentation. The first strand of cDNA was synthesized using random hexamers, and then DNA polymerase I, four dNTPs, and RNaseH were added to synthesize the second cDNA strand. The purified double-stranded cDNA was repaired at the end, A tail was added, and sequencing adapters were connected. The suitable products were selected by agarose gel electrophoresis, and the fragments were PCR amplificated to create a cDNA library. The cDNA library sequencing was conducted with an Illumina high-throughput sequencing platform.

Raw paired-end reads were trimmed and quality controlled by SeqPrep (https://github.com/jstjohn/SeqPrep) and Sickle (https://github.com/najoshi/sickle) programs with default parameters. Next, clean reads were separately aligned to the reference genome of using TopHat v2.0.0 (http://tophat.cbcb.umd.edu/). The mapping criterion of bowtie was as follows: sequencing reads should be uniquely matched to the genome allowing up to two mismatches, without InDels. Expression levels of transcripts were calculated as fragments per kilobase of exon per million mapped reads (FRKM) values via RSEM (http://deweylab.biostat.wisc.edu/rsem/). The clean reads were aligned with the wheat reference genome (http://plants.ensembl.org/Triticum_aestivum/Info/Index) using TopHat 2.0.13 software. Differential (fold-change ≥ 2 and *P* < 0.05) gene expression was determined using the EdgeR (http://www.bioconductor.org/packages/2.12/bioc/html/edgeR.html) software package. Then, the gene ontology (GO) database and the Kyoto Genome Encyclopedia (KEGG) database are used for functional annotation and pathway enrichment of DEGs.

### Reverse transcription-quantitative polymerase chain reaction (RT-qPCR) assays

RT-qPCR was used to examine the relative expression of *TaHMA2b-7A*. After removing genomic DNA using RNase-free DNase I, total RNA was reverse-transcribed into cDNA using the TaKaRa PrimeScript™ RT Reagent Kit Eraser. Then, RT-qPCR was performed with the TaKaRa SYBR®*Premix Ex Taq*™ II on a Bio-Rad C1000 CFX96™ touch Real-time PCR detection system. The thermal regime was as follows: 95ºC for 3 min, 40 cycles of 95ºC for 10 s, and 60ºC for 30 s. The melt curve was plotted using the following melt protocol: 95ºC for 15 s, 60ºC for 1 min, and 60-95ºC for 15 s (+ 0.3ºC/cycle). The relative expression levels of *TaHMA2b-7A* were calculated with the 2^−ΔΔC^_*T*_ method [69] using the publicly reported *TaGAPDH* and *TaActin* as the reference genes [70, 71]. Each sample contained three independent biological replicates.

### Statistical analysis

All data were presented as mean ± standard deviation. Multiple comparisons among different treatments were performed through Student’s t-test or one-way ANOVA using GraphPad Prism 8.0. A value of *P* < 0.05 was considered statistically significant.

## Supplementary Information

**Additional file 1: Supplementary Table S1. **Overview of the transcriptome sequencing data in this study. **SupplementaryFigure S1.** Expression correlation of in shoot and root samples ofwheat plants under salt stress. After7 d of wheat seed germination, the wheat seedlings were transferred to asolution containing 100 mM NaCl for 10 d. The heatmap showed the FPKM values ofgenes. **SupplementaryFigure S2.** The primary metabolic pathway enrichment map of DEGs inthe shoots of wheat plants under salt stress. After 7 d of wheat seedgermination, the wheat seedlings were transferred to a solution containing 100mM NaCl for 10 d. Theheatmap showd the FPKM values of DEG. **SupplementaryFigure S3.** The primary metabolic pathway enrichment map of DEGs inthe roots of wheat plants under salt stress. After 7 d of wheat seed germination,the wheat seedlings were transferred to a solution containing 100 mM NaCl for 10d. Theheatmap showed the FPKM values of DEGs.

## Data Availability

All data generated or analysed during this study are included in this published article [and its supplementary information files]. All the data and materials that are required to reproduce these findings can be shared by contacting Ying-peng Hua (yingpenghua@zzu.edu.cn). All the sequencing data were submitted to the National Centre for Biotechnology Information (NCBI) (http://www.ncbi.nlm.nih.gov/) with the Bioproject of PRJNA744353.

## Abbreviations

DEG: Differentially expressed gene
NHX: Na^+^/H^+^ antiporter
BASS: Bile acid sodium symporter
AKT/KAT: Arabidopsis K^+^ transporter
HKT: High-affinity K^+^ transporter
KEA: K^+^ efflux antiporter
KCO: Two-pore K^+^ channel
SKOR: Stelar K^+^ outward rectifier
ANXD: Annexin D
GLR: Glutamate-like receptor
CAX: Cation exchanger
COPT: Copper transporter
ZIP: ZRT/IRT-related protein
MGT: Magnesium transporter
IRT: Iron transporter
NRAMP: Natural resistance-associated macrophage protein
