# Supplementary material for: Integrated physiological and transcriptional dissection reveals the core genes involving nutrient transport and osmoregulatory substance biosynthesis in allohexaploid wheat seedlings under salt stress
Source: BMC Plant Biol. 2022 Oct 27;22:502. doi: 10.1186/s12870-022-03887-0 (PMC9608917; doi:10.1186/s12870-022-03887-0)
Supplement: Supplementary file 1 — Additional file 1: Supplementary Table S1. Overview of the transcriptome sequencing data in this study. SupplementaryFigure S1. Expression correlation of in shoot and root samples ofwheat plants under salt stress. After7 d of wheat seed germination, the wheat seedlings were transferred to asolution containing 100 mM NaCl for 10 d. The heatmap showed the FPKM values ofgenes. SupplementaryFigure S2. The primary metabolic pathway enrichment map of DEGs inthe shoots of wheat plants under salt stress. After 7 d of wheat seedgermination, the wheat seedlings were transferred to a solution containing 100mM NaCl for 10 d. Theheatmap showd the FPKM values of DEG. SupplementaryFigure S3. The primary metabolic pathway enrichment map of DEGs inthe roots of wheat plants under salt stress. After 7 d of wheat seed germination,the wheat seedlings were transferred to a solution containing 100 mM NaCl for 10d. Theheatmap showed the FPKM values of DEGs. [file 12870_2022_3887_MOESM1_ESM.doc]

**Supplementary Table S1** Overview of the transcriptome sequencing data in this study

| Sample | Raw reads | Raw bases | Clean reads | Clean bases | Mapped rate (%) | Q20  (%) | Q30  (%) | GC content (%) |
| --- | --- | --- | --- | --- | --- | --- | --- | --- |
| CK-S1 | 81,831,040 | 12,356,487,040 | 81,022,372 | 12,060,927,116 | 94.10 | 98.03 | 94.26 | 55.55 |
| CK-S2 | 97,354,592 | 14,700,543,392 | 96,467,486 | 14,382,279,261 | 93.58 | 98.21 | 94.75 | 56.63 |
| CK-S3 | 109,751,026 | 16,572,404,926 | 108,750,122 | 16,179,155,531 | 93.71 | 98.24 | 94.8 | 55.66 |
| NaCl-S1 | 109,751,026 | 16,572,404,926 | 108,750,122 | 16,179,155,531 | 93.83 | 98.24 | 94.8 | 55.66 |
| NaCl-S2 | 94,714,660 | 14,301,913,660 | 93,737,866 | 13,882,995,013 | 93.40 | 98.15 | 94.61 | 56.26 |
| NaCl-S3 | 103,739,288 | 15,664,632,488 | 102,509,840 | 15,165,337,387 | 93.39 | 98.13 | 94.54 | 55.97 |
| CK-R1 | 93,650,802 | 14,141,271,102 | 92,663,246 | 13,671,587,517 | 90.06 | 98.17 | 94.63 | 54.67 |
| CK-R2 | 92,962,154 | 14,037,285,254 | 91,950,192 | 13,546,246,903 | 89.91 | 98.16 | 94.6 | 54.2 |
| CK-R3 | 103,367,072 | 15,608,427,872 | 102,485,512 | 15,264,418,038 | 91.66 | 98.24 | 94.79 | 55.13 |
| NaCl-R1 | 101,859,242 | 15,380,745,542 | 100,781,080 | 14,869,120,475 | 92.15 | 98.11 | 94.46 | 54.84 |
| NaCl-R2 | 95,936,696 | 14,486,441,096 | 94,972,192 | 14,012,091,129 | 92.08 | 98.22 | 94.75 | 54.69 |
| NaCl-R3 | 114,678,738 | 17,316,489,438 | 113,474,588 | 16,643,813,454 | 92.57 | 98.26 | 94.88 | 54.75 |





**Supplementary Figure S1** Expression correlation of in shoot and root samples of wheat plants under salt stress. After 7 d of wheat seed germination, the wheat seedlings were transferred to a solution containing 100 mM NaCl for 10 d. The heatmap showed the FPKM values of genes.


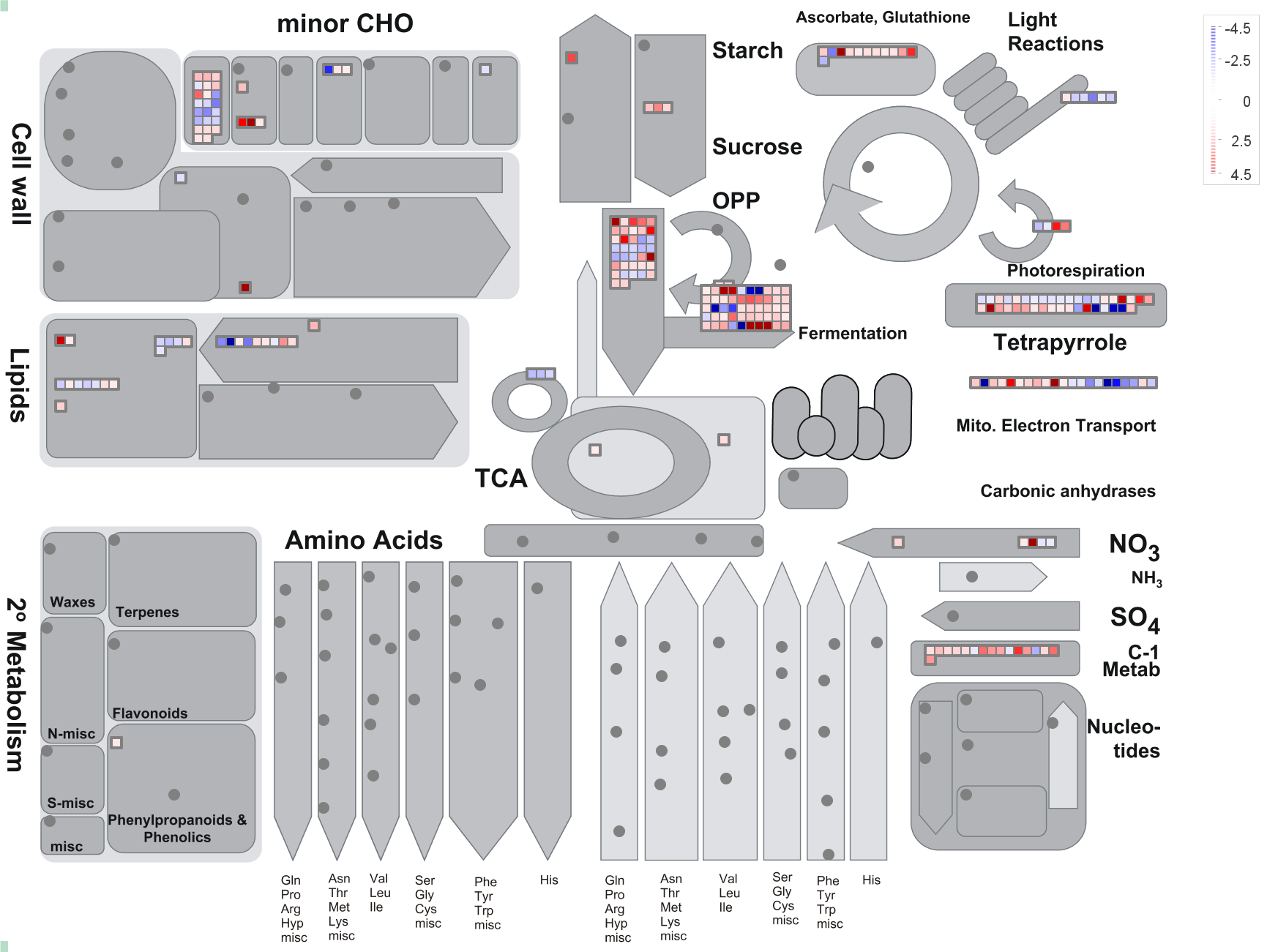


**Supplementary Figure S2** The primary metabolic pathway enrichment map of DEGs in the shoots of wheat plants under salt stress. After 7 d of wheat seed germination, the wheat seedlings were transferred to a solution containing 100 mM NaCl for 10 d. The heatmap showd the FPKM values of DEG.


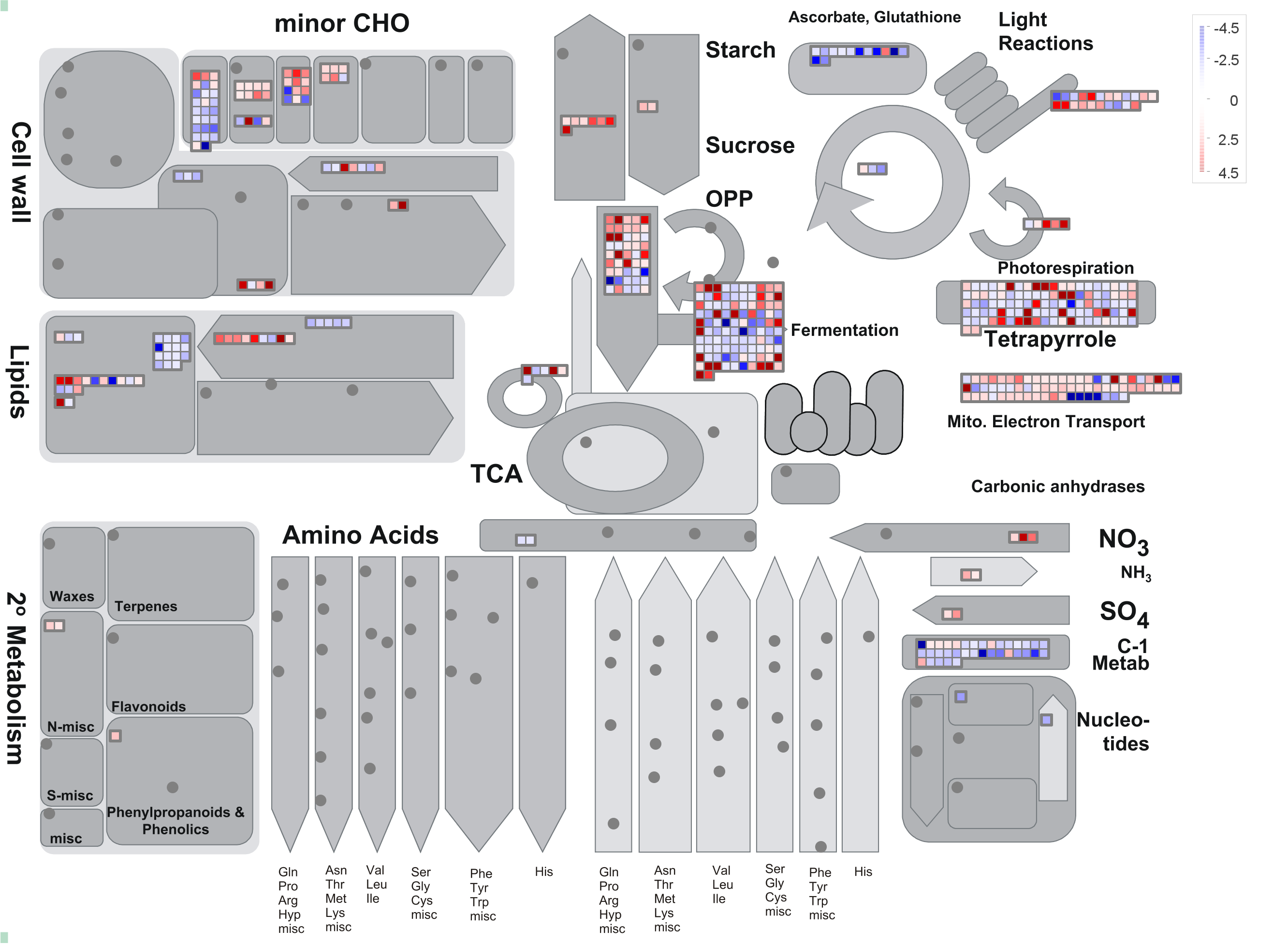


**Supplementary Figure S3** The primary metabolic pathway enrichment map of DEGs in the roots of wheat plants under salt stress. After 7 d of wheat seed germination, the wheat seedlings were transferred to a solution containing 100 mM NaCl for 10 d. The heatmap showed the FPKM values of DEGs.
